# Motion-corrected whole-heart PET-MR for the simultaneous visualisation of coronary artery integrity and myocardial viability: an initial clinical validation

**DOI:** 10.1007/s00259-018-4047-7

**Published:** 2018-05-12

**Authors:** Camila Munoz, Karl P. Kunze, Radhouene Neji, Teresa Vitadello, Christoph Rischpler, René M. Botnar, Stephan G. Nekolla, Claudia Prieto

**Affiliations:** 1 School of Biomedical Engineering and Imaging Sciences, King’s College London, 4th Floor, Lambeth Wing, St Thomas’ Hospital, London, SE1 7EH UK; 2 Nuklearmedizinische Klinik und Poliklinik, Technische Universität München, Munich, Germany; 3 MR Research Collaborations, Siemens Healthcare, Frimley, UK; 4 Escuela de Ingenieria, Pontificia Universidad Catolica de Chile, Santiago, Chile; 5 DZHK (Deutsches Zentrum für Herz-Kreislauf-Forschung e.V.) partner site Munich Heart Alliance, Munich, Germany

**Keywords:** Cardiac PET-MR, Motion correction, Coronary artery disease, Coronary MR angiography

## Abstract

**Purpose:**

Cardiac PET-MR has shown potential for the comprehensive assessment of coronary heart disease. However, image degradation due to physiological motion remains a challenge that could hinder the adoption of this technology in clinical practice. The purpose of this study was to validate a recently proposed respiratory motion-corrected PET-MR framework for the simultaneous visualisation of myocardial viability (^18^F-FDG PET) and coronary artery anatomy (coronary MR angiography, CMRA) in patients with chronic total occlusion (CTO).

**Methods:**

A cohort of 14 patients was scanned with the proposed PET-CMRA framework. PET and CMRA images were reconstructed with and without the proposed motion correction approach for comparison purposes. Metrics of image quality including visible vessel length and sharpness were obtained for CMRA for both the right and left anterior descending coronary arteries (RCA, LAD), and relative increase in ^18^F-FDG PET signal after motion correction for standard 17-segment polar maps was computed. Resulting coronary anatomy by CMRA and myocardial integrity by PET were visually compared against X-ray angiography and conventional Late Gadolinium Enhancement (LGE) MRI, respectively.

**Results:**

Motion correction increased CMRA visible vessel length by 49.9% and 32.6% (RCA, LAD) and vessel sharpness by 12.3% and 18.9% (RCA, LAD) on average compared to uncorrected images. Coronary lumen delineation on motion-corrected CMRA images was in good agreement with X-ray angiography findings. For PET, motion correction resulted in an average 8% increase in ^18^F-FDG signal in the inferior and inferolateral segments of the myocardial wall. An improved delineation of myocardial viability defects and reduced noise in the ^18^F-FDG PET images was observed, improving correspondence to subendocardial LGE-MRI findings compared to uncorrected images.

**Conclusion:**

The feasibility of the PET-CMRA framework for simultaneous cardiac PET-MR imaging in a short and predictable scan time (~11 min) has been demonstrated in 14 patients with CTO. Motion correction increased visible length and sharpness of the coronary arteries by CMRA, and improved delineation of the myocardium by ^18^F-FDG PET, resulting in good agreement with X-ray angiography and LGE-MRI.

## Introduction

Simultaneous PET-MR imaging has shown promising results for the comprehensive assessment of several cardiac conditions in initial clinical studies [1–5]. Dweck et al. [6] observed increased accuracy for the diagnosis of active cardiac sarcoidosis by combining ^18^F-fluorodeoxyglucose (^18^F-FDG) PET and cardiovascular MR (CMR). Nensa et al. [7] showed promising results of increased sensitivity for the detection of borderline myocarditis using hybrid ^18^F-FDG PET and CMR scans. A significant PET-MR intermodality agreement was observed in acute myocardial infarction [8], and more recently CMR in combination with ^18^F-FDG and ^18^F-sodium fluoride (^18^F-NaF) was successfully used for imaging of inflammation and calcification in the coronary arteries [9].

Most of the currently used cardiac PET-MR imaging workflows consist of the acquisition of several MR images with differing contrasts, while simultaneously acquiring list-mode PET data throughout the whole or most of the scanning session. Cardiac MR images acquired in these protocols typically include a stack of cine images, Late Gadolinium Enhancement (LGE), coronary MR angiography (CMRA) and T_1_ and T_2_ mapping, leading to scanning sessions that range between 30 and 90 min in duration [6, 8, 9]. During this prolonged examination time, physiological motion occurs due to breathing and cardiac motion (contraction and relaxation), thus requiring motion compensation techniques to minimise ghosting, blurring and associated image artefacts in both modalities.

Cardiac motion compensation is typically achieved in MR by synchronising the acquisition with an external electrocardiogram (ECG) device and acquiring data only during the quiescent periods of the cardiac cycle, e.g. mid-diastole. Respiratory motion is conventionally addressed in a similar fashion, by acquiring and accepting data only during a specific respiratory phase of minimal motion, typically end-expiration. Surrogate signals that have a strong correlation with the respiratory motion of the heart are used for synchronisation, either from external devices (such as respiratory bellows) or image-derived signals (such as monitoring the position of the lung-liver interface, so-called 1D image navigators) [10]. These approaches, however, lead to long and unpredictable scan times due to breathing pattern variations and drifting. Similar approaches have been proposed for cardiac PET imaging [11], so that the acquired list-mode data are dual-gated into a number of cardiac and respiratory phases, and only data acquired during end-expiration and mid-diastole are used for image reconstruction. However, the significant reduction in data available in each gate produces images with increased noise. Therefore, in practice, most of the clinical studies still aggregate all the acquired data into a single static time frame blurred by both respiratory and cardiac motion [6, 8].

Simultaneous PET-MR technology has shown potential for addressing the motion problem in PET by incorporating motion information estimated from simultaneously acquired MR images into PET image reconstruction. In order to obtain motion information, a set of MR images is acquired at different cardiac and respiratory positions, and motion is estimated by non-rigid registration of the images for each frame, typically using the end-diastolic and end-expiration frame as reference [12]. The simultaneously acquired PET data is then sorted into corresponding frames, and MR-based motion fields are incorporated into the PET image reconstruction [13, 14] to reduce motion induced blurring and artefacts, improving PET image quality and quantification [12].

In most of the current approaches for MR-based motion correction of cardiac PET data, the MR images acquired simultaneously with PET are designed for motion estimation purposes only [15–17]. The lack of appropriate tissue contrast and/or sufficient spatial resolution of these MR images might prevent their use for diagnostic purposes. This may lead to prolonged examination times, since diagnostic MR images are to be acquired after the simultaneous PET-MR examination.

We have recently proposed a different approach for the acquisition of motion-corrected cardiac PET-MR images [18]. With this approach motion estimated from MR images is used to correct both ^18^F-FDG PET and CMRA datasets, enabling visualisation of myocardial viability and coronary anatomy in a single efficient examination. This PET-CMRA motion correction framework is based on 2D image-based navigation [19, 20], thus allowing for 100% scan efficiency (no respiratory gating window with concomitant data rejection) and predictable scan time. The feasibility of this PET-CMRA framework was shown in a small cohort (*N* = 5) of oncology patients without known or suspected coronary artery disease (CAD) [18], however its feasibility in patients with cardiovascular disease has not been investigated yet. Here we present an initial clinical validation of this method for the simultaneous visualisation of myocardial integrity by ^18^F-FDG PET and coronary lumen integrity by CMRA. The study was performed in a cohort of patients with known CAD, in particular, patients with chronic total occlusion of at least one of the coronary arteries.

## Materials and methods

### PET-MR image acquisition

**Fig. 1:**
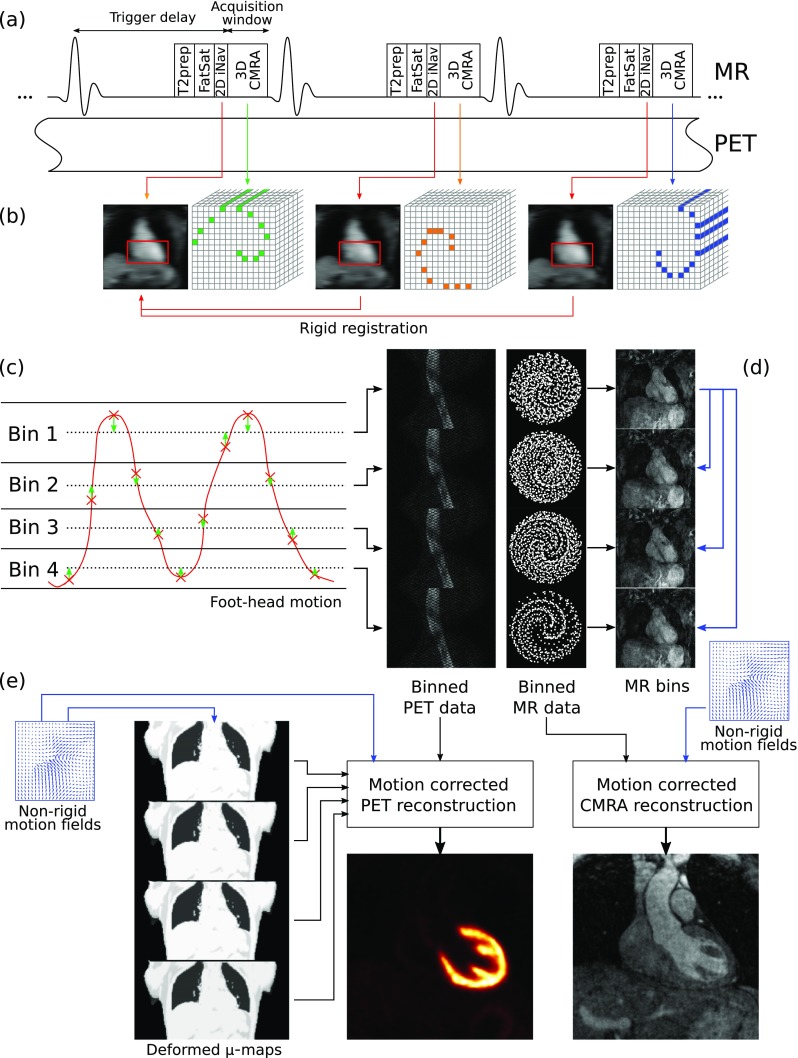
PET-CMRA acquisition and reconstruction scheme. **a** PET-CMRA acquisition sequence, an image-navigator (2D iNAV) is acquired at each heartbeat before the CMRA acquisition for estimating respiratory motion. T2prep and FatSat preparation pulses are performed to improve contrast between blood and surrounding tissue. **b** Translational motion in FH and RL directions is estimated from the 2D iNAVs by rigid registration of a template covering the apex of the heart (in *red*). **c** FH motion is used to bin the PET and CMRA data in a number of respiratory windows. FH and RL motion is used to correct the CMRA data to the centre of each bin (*green arrows*). **d** Non-rigid deformation fields are estimated from MR images reconstructed at each respiratory position and **e** used to move the attenuation maps to each respiratory position, and in motion compensated PET and CMRA image reconstruction

The whole-heart PET-CMRA acquisition consists of a free-breathing ECG-triggered CMRA sequence simultaneously acquired with list-mode cardiac PET data (Fig. 1a) as proposed in [18]. CMRA data is acquired using a 3D spoiled gradient echo sequence following a fully sampled golden-step Cartesian trajectory with spiral profile ordering [21], so that one spiral interleaf is acquired at each heartbeat. A low-resolution coronal 2D image navigator (iNAV) [19] for respiratory motion estimation is acquired at the beginning of each interleaf by spatially encoding low flip angle k-space lines. Fat saturation (Fat Sat) and T2-preparation (T2-prep) [22] pulses are performed immediately prior to data acquisition to improve the contrast between arterial blood and the surrounding myocardium and epicardial fat.

As part of the cardiac PET-CMRA acquisition protocol, a standard Dixon-based attenuation map (μ-map) is acquired in breath-hold at end-expiration for MR-based attenuation correction of the PET data [23], with missing tissue due to the limited field of view of the MR (as compared to PET) estimated using the MLAA (Maximum Likelihood reconstruction of Attenuation and Activity) approach [24]. Additionally, a conventional 2D cine image is acquired for defining the trigger delay and length of the acquisition window of the 3D CMRA.

### PET-MR image reconstruction

Motion compensated image reconstruction is performed in four steps (Fig. 1b-e). In the first step, foot-head (FH) and right-left (RL) translational respiratory motion is estimated from the 2D iNAVs by using rigid image registration of a template covering the apex of the heart (Fig. 1b). This allows measuring directly the respiratory-induced translational motion of the heart in a beat-to-beat fashion. In the second step, FH motion is used to bin the acquired PET and CMRA data in a number of respiratory windows ranging from end-expiration to end-inspiration, each containing the same amount of data (Fig. 1c). Outlier rejection is performed in this step, by excluding CMRA data acquired in deep breaths [18]. CMRA data acquired within each respiratory window is then corrected to the centre of the bin by applying a phase shift in k-space according to the estimated FH and RL motion. In a third step, 3D MR images are reconstructed at each respiratory position using iterative SENSE with a soft-binning approach [18, 20], and bin-to-bin respiratory deformation fields are estimated by non-rigid image registration, using the end-expiration bin as reference (Fig. 1d). Finally, the non-rigid deformation fields are used in a generalised matrix description formulation for motion-compensated CMRA reconstruction [25]. Moreover, the non-rigid motion fields are also used to move the attenuation maps to each respiratory position (Fig. 1e) and perform a motion-compensated PET reconstruction. Therefore, at the end of the reconstruction process, co-registered respiratory motion-corrected CMRA and cardiac PET images are obtained.

### Experiments

**Fig. 2:**
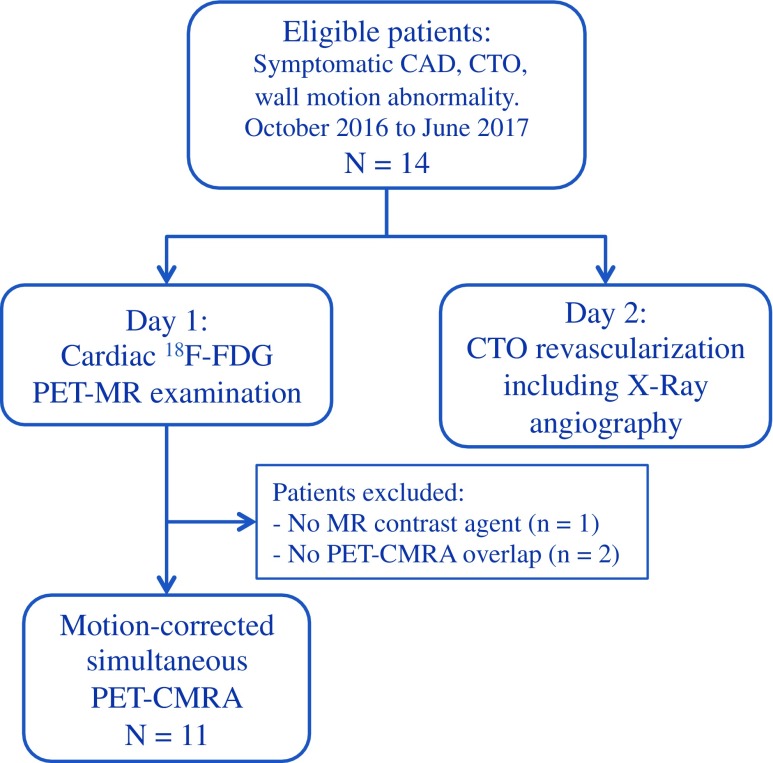
Flow chart of patients included in the study

Fourteen patients with symptomatic CAD (angina or angina equivalent, excluding acute STEMI patients), chronic total occlusion (CTO) of a relevant coronary artery (segment 1, 2, 6, 7, 11 or 13, diameter > 2.5 mm), and echocardiographic or angiographic evidence of a wall motion abnormality in the corresponding area were recruited for this study between October 12, 2016 and June 26, 2017 (Fig. 2). In order to improve risk stratification before elective Percutaneous Coronary Intervention (PCI) of the CTO, all patients underwent a hybrid ^18^F-FDG PET-MR examination in a Biograph mMR scanner (Siemens Healthcare, Erlangen, Germany). Relevant patient characteristics include: age 66.1 ± 9.5 years, 9 males, 5 females, LVEF 49 ± 12, and previous PCI stenting of at least one of the coronary arteries. Written informed consent with respect to participation was obtained from all subjects; the study was performed in concordance with the Declaration of Helsinki and approved by the institutional ethics committee.

The clinical PET-MR examination protocol included a scan of 40 to 50 min with a list-mode PET acquisition for the assessment of myocardial viability under insulin-clamped conditions 60 min after injection of 326 ± 29 MBq of ^18^F-FDG, and a multi-slice 2D-Phase Sensitive Inversion Recovery (PSIR) LGE acquisition (1.4–2.2 mm in-plane resolution, 8 mm slice thickness). During the 10–15 min waiting time required for optimal contrast in LGE images, an acquisition with a prototype implementation of the proposed PET-CMRA sequence was performed.

For the CMRA acquisition, relevant imaging parameters included: coronal orientation, resolution = 1 × 1 × 2 mm^3^ (interpolated to 1mm^3^ isotropic resolution during image reconstruction), field of view = 304 × 304 × 88–112 mm^3^ covering the whole heart, TR/TE = 3.72/1.70 ms, and flip angle = 15°. A subject-specific trigger delay was defined targeting the mid-diastolic quiescent period of the cardiac cycle, and an acquisition window of 89 to 119 ms (corresponding to 24 to 32 lines per spiral interleaf) was selected depending on the length of the mid-diastolic period. The 2D iNAVs were acquired using the following parameters: same field of view as the CMRA acquisition, flip-angle = 3°, 14 acquired lines with a centric-in Cartesian trajectory, corresponding to a 1 × 21.7 mm^2^ acquired resolution, interpolated to 1 × 1 mm^2^ before FH and RL motion estimation. The vendor-provided Fat Sat prepulse was used, and an adiabatic T2-prep (50 ms duration) was implemented to improve tissue contrast. μ-maps were acquired for each patient during a 19 s breath-hold at end-expiration using the vendor’s standard Dixon protocol (acquisition parameters: coronal orientation, resolution = 2.6 × 2.6 × 3.1 mm^3^, field of view = 328 × 500 × 399 mm^3^, TR/TE1/TE2 = 3.60/1.23/2.46 ms). All patients underwent interventional X-ray angiography the day after the PET-MR examination, for elective CTO revascularization.

CMRA and PET datasets were reconstructed with the described motion correction scheme (MC) and without motion correction (NMC) for comparison purposes. For each patient, the fraction of list-mode PET data acquired simultaneously with the CMRA sequence was selected for PET image reconstruction. Due to variations in planning time, the overlap between CMRA and list-mode PET acquisition varied between patients, with an average overlap of 80.3 ± 20.9% of the duration of the CMRA acquisition.

MR image reconstruction was performed offline in MATLAB (Mathworks, Natick, Massachusetts, USA) using custom developed software. PET image reconstruction was performed offline using reconstruct-transform-average [13] motion correction. For this, the standard μ-map acquired at end-expiration was moved to each respiratory position in MATLAB using the deformation fields estimated from MR images. Each respiratory bin was independently reconstructed offline with e7 Tools (Siemens Healthcare, Knoxville, USA) using the OSEM algorithm, with three iterations and 21 subsets, point spread function modelling, voxel size = 2.03 × 2.08 × 2.08 mm^3^, and matrix size = 127 × 344 × 344. Finally, images reconstructed at each respiratory position were combined in MATLAB to produce a motion corrected PET image.

### Image analysis

Reconstructed CMRA images were reformatted to simultaneously visualise the left anterior descending (LAD) artery and right coronary artery (RCA) using dedicated software [26]. Metrics of visible vessel length and sharpness were obtained for vessels without stents in the proximal artery. Vessel sharpness values were normalised to the signal intensity of the centre line of each vessel, so that 100% sharpness refers to a maximum signal intensity change at the vessel edge. Additionally, motion corrected CMRA images were reformatted following the anatomy observed in the X-ray angiography in order to compare both visually.

Reconstructed ^18^F-FDG PET images were analysed by automatically applying the 17-segment model according to the American Heart Association [27] to the left ventricular myocardium using dedicated software (MunichHeart [28]). The relative increase in ^18^F-FDG signal of motion corrected images over uncorrected images was computed for each of the 17 segments for each patient. Additionally, NMC and MC PET images were reoriented in short axis to visually compare them with LGE images.

## Results

Scans were successfully completed in all subjects. The average acquisition time for the proposed PET-CMRA framework was 11.2 ± 2.4 min. Due to differences in planning time, in two of the patients there was no overlap between the PET and the CMRA acquisition, so that motion correction of the PET data could not be performed. Additionally, one of the patients was not able to receive the gadolinium-based contrast agent. These three patients were excluded from the following analysis. For the rest of the patients (*N* = 11), the average overlap between PET and CMRA acquisition was 8.8 ± 1.2 min, corresponding to list-mode PET data being acquired during 80.3 ± 20.9% of the duration of the CMRA acquisition on average.

**Fig. 3:**
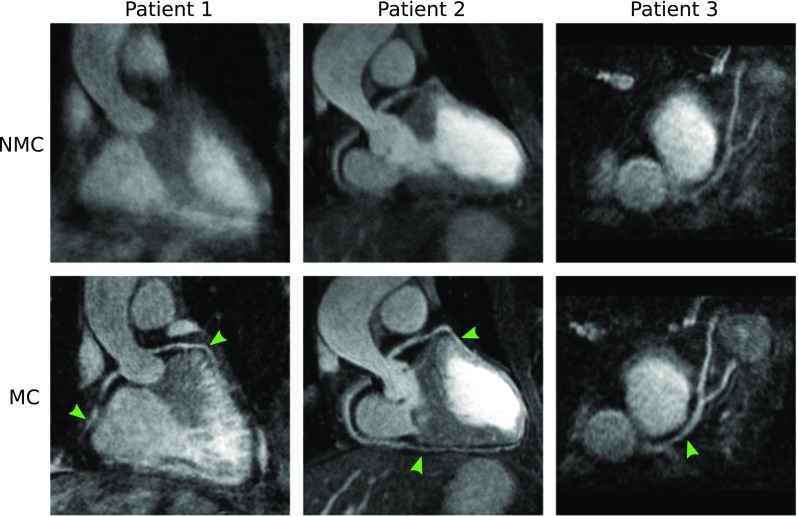
Reformatted images for three representative patients (*columns*) showing uncorrected (NMC) and motion-corrected (MC) CMRA. Improvements in the visualisation of the vessels are observed when applying MC (*green arrows*) for all cases, particularly in the distal segments of the arteries

**Fig. 4:**
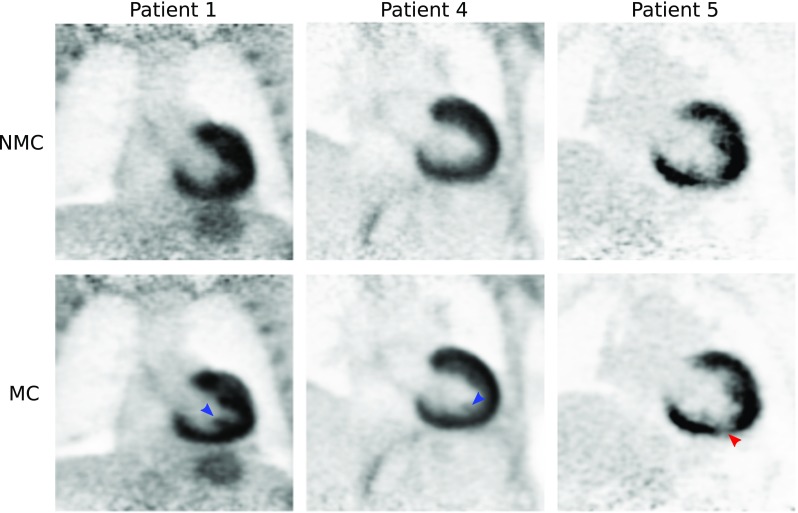
Coronal slice for three representative patients (*columns*) showing uncorrected (NMC) and motion-corrected (MC) viability ^18^F-FDG PET images. Improvements in image quality can be observed when applying MC, particularly in small structures (*blue and red arrows*), compared to NMC

Reformatted images showing non-stented RCA and LAD for three of the patients are shown in Fig. 3. Improvements in the delineation of the RCA and LAD were observed in the CMRA images after applying MC, allowing for depiction of non-stented vessels, even in cases where severe respiratory motion prevented the visualisation of both the left and right proximal coronary arteries in the NMC image, as observed for Patient 1. Figure 4 shows example coronal slices of the ^18^F-FDG PET images reconstructed for three representative patients. MC increased the sharpness of the left ventricle myocardium and improved visualisation of small features such as the papillary muscles (blue arrows) and delineation of viability defects (red arrow) compared to NMC.

**Fig. 5:**
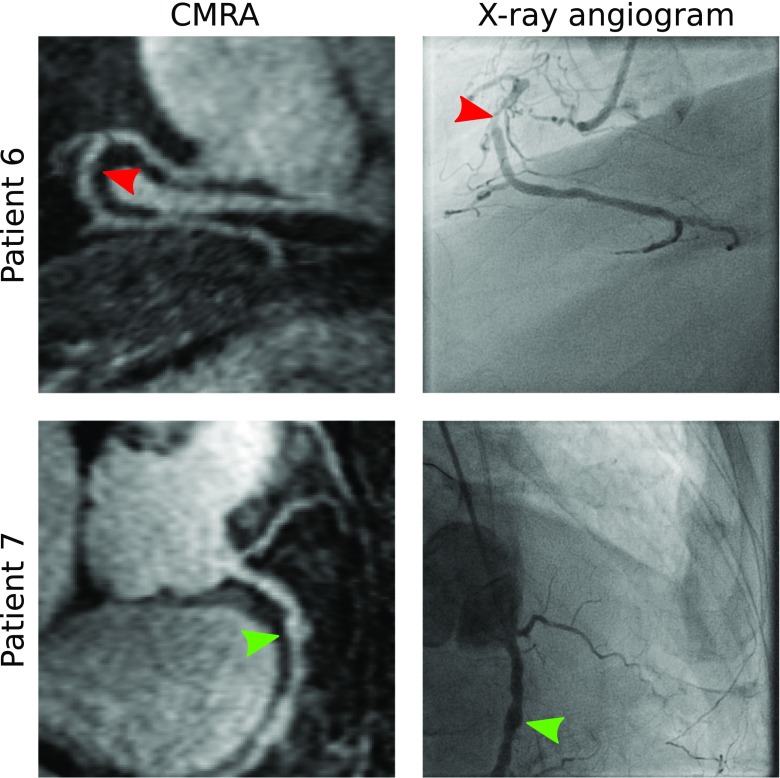
Reformatted CMRA and corresponding X-ray angiogram for two representative patients. *Red arrows*: stenosis in the mid segment of the right coronary artery (RCA); *green arrows*: aneurysm in the proximal segment of the RCA

A visual comparison between the reformatted MC CMRA and the corresponding invasive X-ray angiogram is shown in Fig. 5 for two patients. Adequate spatial resolution and contrast in CMRA images allowed for a depiction of the proximal arteries comparable to the X-ray angiogram for both cases. In Patient 6, a stenosis observed in the mid segment of the RCA in CMRA was confirmed in the angiogram (Fig. 5, *red arrows*), while in Patient 7 an aneurysm in the proximal RCA was seen in both modalities (Fig. 5, *green arrows*).

**Fig. 6:**
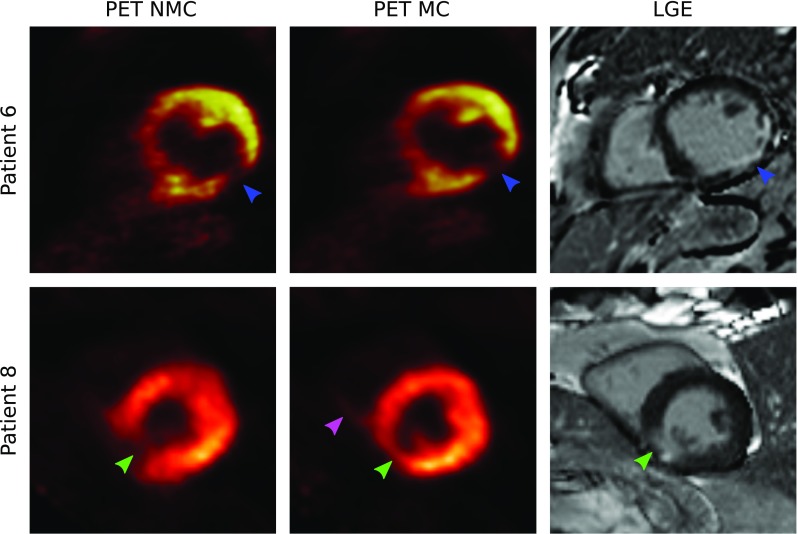
Short axis view for two representative patients (*rows*) showing uncorrected (NMC) and motion-corrected (MC) viability ^18^F-FDG PET images, and corresponding 2D LGE scans. MC improves the correspondence of the PET images to the anatomy as observed in the LGE images, particularly in the delineation of viability defects (*green and blue arrows*) and right ventricle myocardium (*pink arrow*)

Figure 6 shows a short axis view of the ^18^F-FDG PET both for NMC and MC reconstructions and corresponding slice of the 2D LGE scan for two patients. It can be observed that MC PET images have an improved correspondence to the anatomy as observed in the LGE images and reduced noise compared to NMC images. In particular, improvements in delineation of viability defects are apparent: for Patient 6, the transmural viability defect observed in the infero-lateral wall was better depicted after motion correction (Fig. 6, *blue arrows*), while in Patient 8, motion correction allowed for the identification of viable myocardium in a defect that appeared misleadingly as transmural in the NMC image (Fig. 6, *green arrows*). MC also enabled the depiction of thinner structures, such as right ventricle myocardium (Fig. 6, *pink arrow*).

**Fig. 7:**
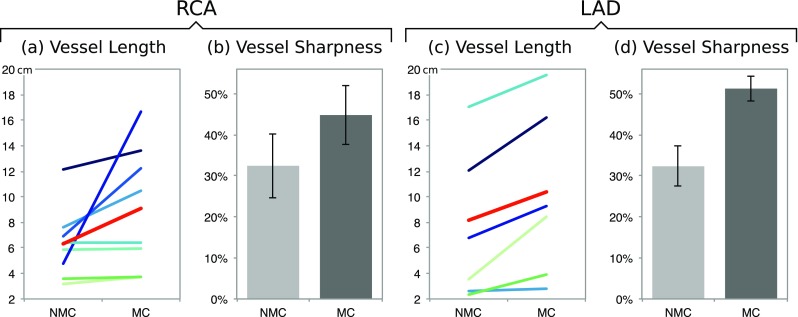
Image quality analysis for uncorrected (NMC) and motion-corrected (MC) CMRA images in 11 patients, including: visible vessel length for (**a**) RCA and (**c**) LAD, for each of the patients (*green/blue lines*) and average (*red line*); and vessel sharpness for (**b**) RCA and (**d**) LAD. Visible vessel length and sharpness improve after motion correction for all cases. Image analysis was performed in vessels without stents in the proximal segment

Image quality metrics for the NMC and MC CMRA images are shown in Fig. 7. Tracking of the vessels was possible in 14 out of 15 non-stented vessels (8 RCA, 6 LAD). In one of the patients, significant cardiac motion prevented the visualisation of the coronary arteries. For the rest of the vessels, increased visible length of the vessels were observed after MC for both the RCA (+49.9% on average) and the LAD (+32.6% on average) (Fig. 7a, c). Similarly, vessel sharpness increased by 12.3% and 18.9% on average for the proximal RCA and LAD respectively when using MC (Fig. 7b, d).

**Fig. 8:**
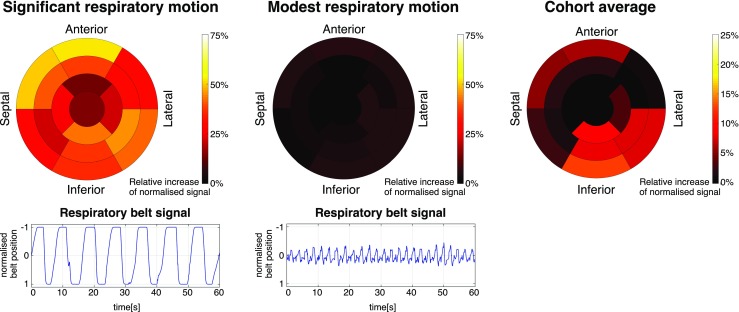
17-segment polar maps of the relative increase in ^18^F-FDG PET signal after motion correction for the left ventricular myocardium. Representative patients with (**a**) high amplitude respiratory motion and (**b**) moderate respiratory motion, and (**c**) the average across the cohort

Polar maps showing the relative increase in ^18^F-FDG PET signal after motion correction in the left ventricular myocardium are displayed in Fig. 8 for two patients with distinct respiratory patterns (Fig. 8a-b) and on average across the cohort (Fig. 8c). For the patient with significant respiratory motion (Fig. 8a), the average signal increased across all segments was 33%, with a maximum in the anterior wall. For the patient with moderate respiratory motion (Fig. 8b), an average of 3% signal increase was observed across all segments. When considering the cohort averages for each myocardial segment (Fig. 8c), signal increased most towards the inferior and inferolateral regions, i.e. by an average of 8% across the six corresponding sectors of the myocardium.

**Fig. 9:**
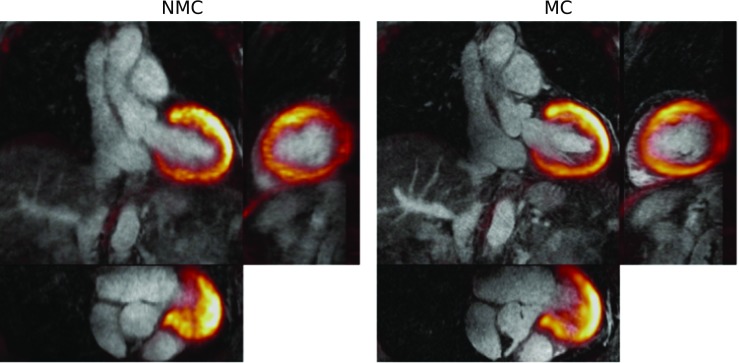
Example fused PET-CMRA image (Patient 4) showing uncorrected (NMC) and motion-corrected (MC) images. The motion correction framework produces co-registered diagnostic PET and MR images where an improved correspondence between both modalities can be observed

Figure 9 shows an example fused PET-CMRA dataset before and after motion correction. The framework produces co-registered diagnostic PET and CMRA images, improving the correspondence between modalities compared to uncorrected images.

## Discussion

In this study we have tested a novel approach for the simultaneous visualisation of coronary anatomy by CMRA and myocardial viability by ^18^F-FDG PET in patients with coronary artery disease. The framework incorporates 2D image-navigation, and uses motion estimated from MR to correct both the PET and CMRA datasets, producing respiratory motion-corrected co-registered images. In contrast to recently proposed MR-based motion correction approaches for cardiac PET data that utilise MR images mainly for improving PET image quality [15–17], our approach produces diagnostic images in both modalities, potentially reducing total examination time. Furthermore, the proposed PET-CMRA acquisition and reconstruction scheme has a short and predictable scan time of approximately 11 min, which makes it suitable for clinical practice. Indeed, for this study the proposed PET-CMRA acquisition was included into a standard clinical PET-MR protocol during the waiting time required for conventional LGE imaging.

The motion correction framework for CMRA data has been previously shown and validated for healthy subjects [18, 20], but to the best of the authors’ knowledge, this is the first study that shows the feasibility of applying such framework to patients with cardiovascular disease. For the cohort of patients in this study, improvement in image quality after motion correction is apparent, allowing for the visualisation of 14 out of 15 non-stented vessels and increasing visible length and sharpness of the coronary arteries. Furthermore, visual comparison showed good agreement between the motion-corrected CMRA and gold standard invasive X-ray angiograms, including visualisation of stenosis and aneurysm in the proximal segment of the arteries (Fig. 5).

For the ^18^F-FDG myocardial viability PET images, the proposed motion correction approach improved the depiction of small structures such as the right ventricle myocardium and papillary muscles, and enhanced the visualisation of transmural and non-transmural viability defects in the left ventricle myocardium. In general, motion correction produced an increased PET signal in each of the myocardial segments, with an average of 8% relative increase for the inferolateral wall. When analysing the relative signal increase for each dataset, it was observed that improvements were strongly related to the respiratory pattern of each subject: modest increase in signal was obtained in patients with shallow breathing, while increases of up to 33% on average were observed in patients with significant respiratory motion. Such increases come both from a reduction in the blurring and from appropriate alignment between attenuation maps and emission data in the motion corrected reconstruction framework. This is particularly significant in the anterior wall (Fig. 8a), located closer to the heart-lung interface, which is consistent with findings in recent studies [29]. Our results suggest that respiratory motion compensation is fundamental for avoiding misinterpretation of non-viable segments in the myocardium by ^18^F-FDG PET. An improved correspondence to the anatomy and delineation of transmural and non-transmural myocardial viability defects was observed when comparing LGE-MRI with the motion corrected PET images. Recent studies have compared conventional LGE-MRI and ^18^F-FDG PET for the assessment of myocardial viability [8, 30], finding good agreement between the two modalities. However, both studies report some mismatches between ^18^F-FDG PET and LGE. It is worth noting that in these studies LGE-MR images were acquired during breath-hold using 2D ECG-triggered sequences, as conventionally done in clinical practice, to minimise cardiac and respiratory motion. On the contrary, in both studies, PET images did not include any motion compensation technique, which could partially explain the mismatches between the two modalities. Further simultaneous ^18^F-FDG PET and LGE-MRI studies with 3D MR acquisition sequences that allow for motion compensation in both modalities (such as the recently proposed BOOST sequence [31]) could allow for a fairer comparison.

This study has a number of limitations. First, the presence of stents in at least one of the coronary arteries for each of the patients limited the number of MR visible vessels available for analysis. A larger cohort would be required to provide a sufficient number of vessels for a more robust statistical analysis of improvements in image quality after motion correction. Second, variations in the overlap between the CMRA and PET acquisitions resulted in high variability in the fraction of PET data that could be motion corrected and therefore in the quality of the PET images. An acquisition protocol with an extended PET list-mode acquisition that guarantees full overlap between the two acquisitions would allow for using the full capabilities of the motion correction approach. Third, patients with known chronic total occlusion were recruited for this study. In order to assess the diagnostic performance of the proposed simultaneous visualisation of myocardial viability and coronary anatomy a similar study would need to be performed in subjects with suspected coronary artery disease.

Our PET-CMRA protocol includes the acquisition of a Dixon-based attenuation correction map, which segments the image into four tissue classes and assigns a fixed attenuation value to each of them [23]. Studies have shown that assigning a fixed attenuation value to the lung tissue can affect accuracy of the PET images in the thoracic region, inducing bias in quantification of lung lesions [32] and volumes of interest within the lungs [33]. However, such effect was shown to be less significant in cardiac structures. Moreover, the study by Lau et al. [34] showed no statistically significant difference in average myocardial uptake when comparing PET-CT and PET-MR using Dixon-based attenuation correction.

The proposed motion compensation technique only addresses the problem of respiratory motion. In CMRA, the problem of cardiac motion was addressed by acquiring data only during the quiescent mid-diastolic period of the cardiac cycle. A similar approach could be used for the PET data by rejecting the fraction of data that falls outside the diastolic period, however, this would impact image quality by increasing the noise in the PET images. Alternatively, if information about cardiac motion is available, it could be included in the PET image reconstruction process [35]. The proposed approach does not include a mechanism for compensating for unpredictable bulk motion of the patient during the PET-CMRA examination. Techniques such as the one proposed by Kolbitsch et al. [36] for detection and correction of whole-body motion in both PET and MR could be adapted to be used with the proposed framework. Finally, in this work, one PET image is independently reconstructed for each respiratory position, and then transformed into the reference end-expiratory position. This approach has been shown to introduce bias due to the reduced number of counts in each respiratory position in simulation studies [37, 38]. Future work will include the deformation fields directly in the motion compensated PET reconstruction process by modifying the system matrix [14].

## Conclusion

We have presented a first clinical validation of a novel respiratory motion-corrected whole-heart PET-CMRA framework for simultaneous visualisation of coronary anatomy and myocardial integrity in patients with coronary artery disease. The framework allows for acquiring diagnostic images with both modalities in a time efficient examination. The reported results have shown that motion correction improves image quality for both modalities compared to the uncorrected images. A good agreement between coronary anatomy depicted by motion-corrected CMRA and X-ray angiography was observed. In addition, motion-corrected ^18^F-FDG PET images were in good agreement with LGE-MRI, showing more accurate depiction of both transmural and non-transmural viability defects.
